# Misleading Diagnostics After a Presumed Recovery of Gallstone Spillage

**DOI:** 10.26502/jsr.10020084

**Published:** 2020-08-31

**Authors:** M Arbogast, C A Nebiker

**Affiliations:** Department of Visceral Surgery, Kantonsspital Aarau, Switzerland

## Introduction

Laparoscopic cholecystectomy is a routine, gold standard treatment for acute cholecystitis and symptomatic cholecystolithiasis. Gallbladder perforation and bile leakage, as well as gallstone spillage, are frequently observed during surgery, with a rate of up to 40%. In case of intraoperative gallbladder perforation, gallstones can get lost, leading to intra-abdominal, abdominal wall or surgical site abscess formation. Such complications can result in diffuse abdominal pain, which are challenging to diagnose.

## Case Report

A fit 63-year old man in presented to our institution with a painful swelling in his right flank after a long hike. His past medical history was unremarkable apart from a laparoscopic cholecystectomy after recurrent episodes of cholecystitis. Our working diagnosis was a lumbar abscess beneath a skin callus secondary to friction from the backpack he was wearing. We intended to drain the abscess but were not able to localize a pus collection. We therefore proceeded to an ultrasound-guided fine needle aspiration for further diagnosis of the suspected fluid collection. We were able to aspirate the entire perifascial fluid collection and the patient reported symptom relief shortly thereafter. Microbiological investigations were positive for Hafnia alvei, part of the normal gastrointestinal flora. We extended our search for a focus of infection, such as diverticulitis with retroperitoneal fistula for example, however, the colonoscopy was normal. An outpatient MRI scan of the abdomen and lumbar spine was reported to show a Petit’s hernia or a sequestered hematoma extending from the paracolic gutter through the retroperitoneum into the lumbar fatty tissue. More than a month later, the patient was re-admitted with the same symptoms of swelling and pain in the right lumbar region. On examination, a seven centimeter fluctuant swelling was palpable.

**Figure 1:**
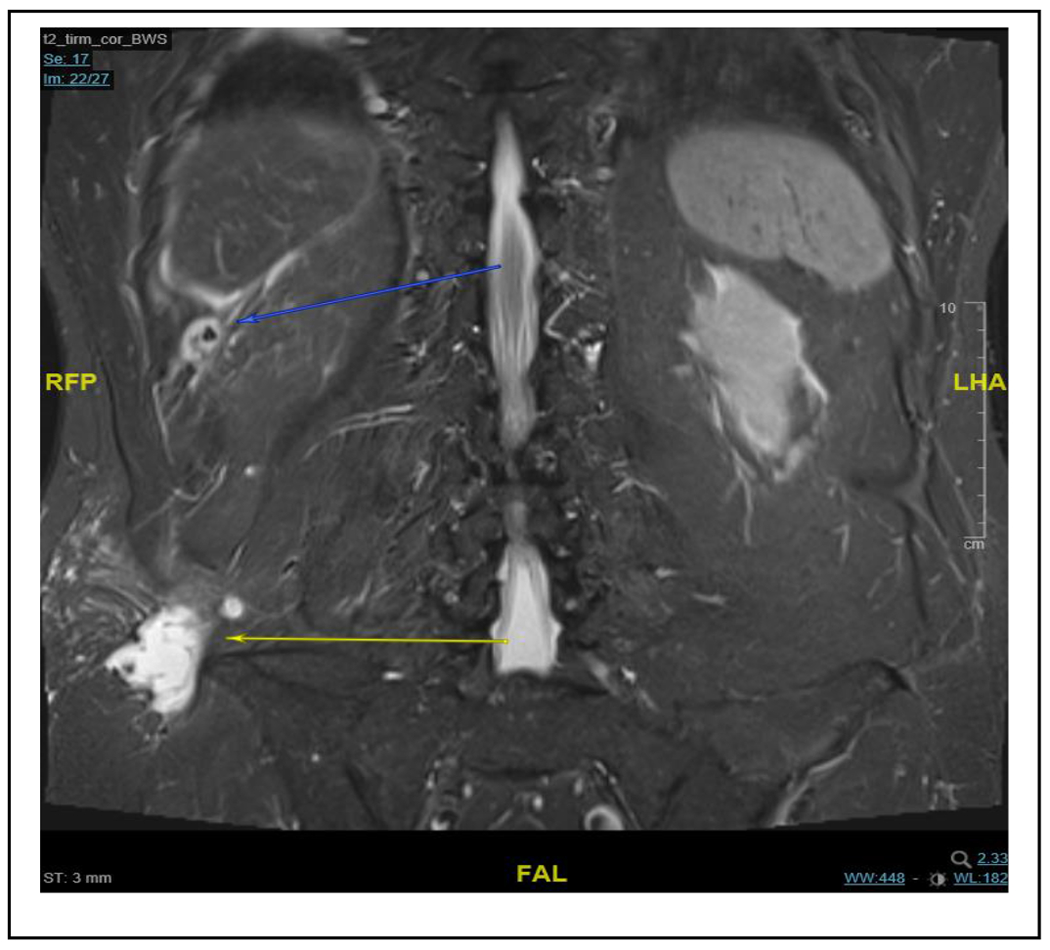
The coronal plane of the MRI scan shows a hyperintense and central hypointense formation caudal to the liver (blue arrow), adjacent to a septated collection alongside the paracolic gutter forming a hyperintense region cranially of the right iliac crest extending dorsally through the lumbar triangle (yellow arrow).

**Figure 2:**
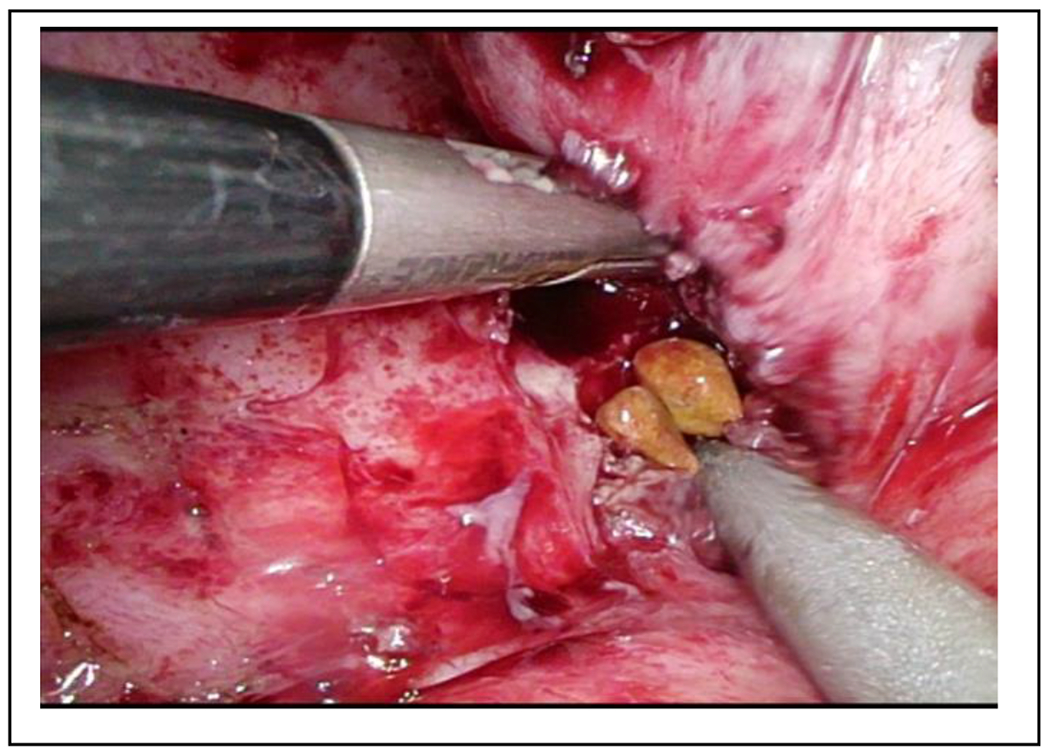
Laparoscopic view showing encapsulated retained gallstones just underneath liver segment VI adjacent to the right colonic flexure.

**Figure 3:**
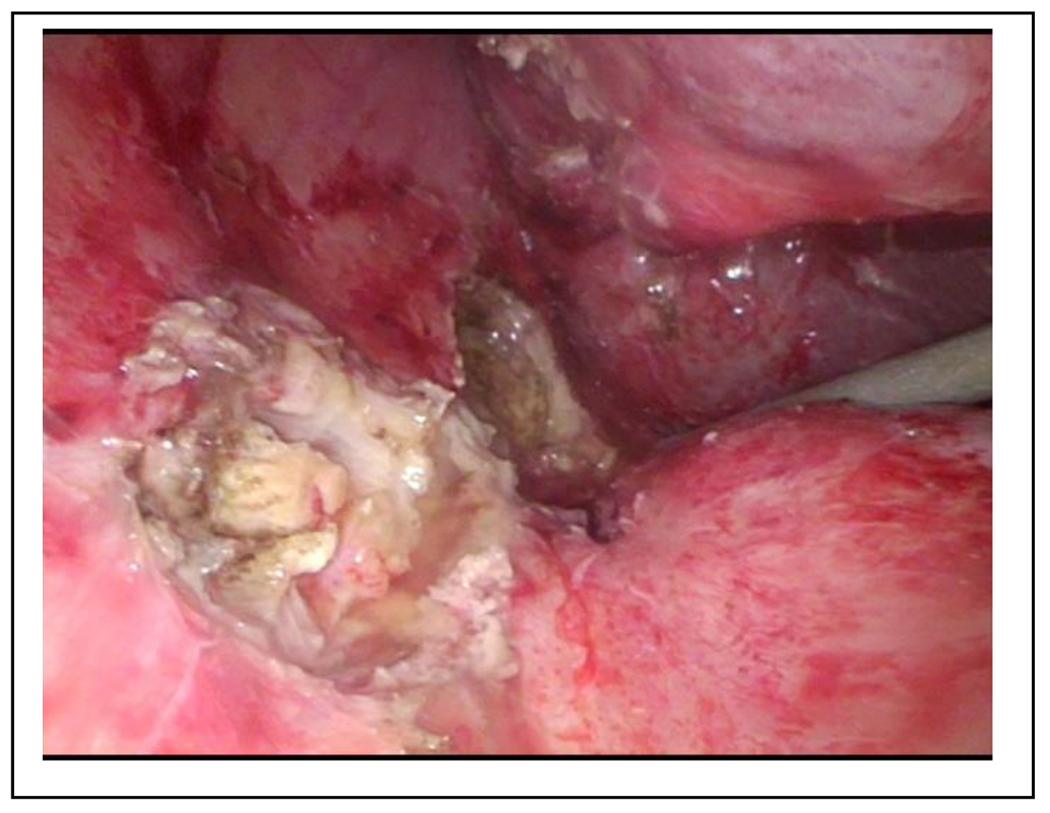
Laparoscopic view showing the right paracolic gutter after carefully exploring sequestered pouches, containing inflammatory exudate and gallstone fragments, within the abdominal wall and retro peritoneum.

With a clinically confirmed lumbar abscess, we again prepped the patient for surgical debridement. This time, we found a foreign body, in the subcutaneous tissue, resembling a gallstone. In addition, a course of antibiotic therapy with cefuroxime was administered. Further evaluation of the MRI and additional CT scans led to the suspicion of a retroperitoneal abscess secondary to missed abdominal gallstones (Figure 1). This hypothesis was supported by the surgical report from the initial laparoscopic cholecystectomy, which described an iatrogenic gallbladder perforation with gallstone spillage but complete collection. We postulated missed gallstone fragments and performed an explorative laparoscopy. As expected, we were able to retrieve four gallstones within this partially sequestered perihepatic abscess (Figure 2). Furthermore, we detected fibrinous plaques and erosions alongside the right paracolic gutter, which we cleaned by thorough irrigation of the abdominal cavity (Figure 3). The postoperative course was straightforward and the patient was discharged after eight days. Three weeks postoperatively, another gallstone passed through the retroperitoneal fistula and out through the lumbar wound. The further recovery process was uneventful and showed complete wound healing.

## Discussion

This case represents the difficulty in connecting a patient’s past history of gallbladder removal to a current retroperitoneal, subcutaneous lumbar abscess. Previously reported subcutaneous swellings or abscess formation caused by ectopic gallstones in the abdominal wall have mostly been connected to laparoscopic port or percutaneous port sites, [3] whereas our report shows an unusual complication with a challenging series of diagnostic investigations. Although many reports of postoperative complications caused by retained gallstones have been published, here we describe the formation of a retroperitoneal transcutaneous lumbar abscess. It originated from one of the less common sites of infection: the paracolic gutter. Most often, infections from retained gallstones occur in the subhepatic space (34.1%), followed by subphrenic (15.9%) and ovarian abscesses (11.4%) according to Akhtar, Nukhari M M, Tariq U, et al [1]. Furthermore, this rare case illustrates a late complication, which at first sight didn’t seem to have an anatomical correlation with former surgery.

In addition, the microbiological results showing Hafnia alvei in the drained abscess misled us when searching for an infectious origin of the retroperitoneal abscess. Usually intra-abdominal abscesses caused by iatrogenic gallbladder perforation or retained gallbladder stones, most often contain the same flora as an infected gallbladder, such as Escherichia coli [2]. Therefore, we first linked our microbiological findings to the intestinal flora of the colon. Retrospectively, considering the extensive diagnostic tests performed, we should have paid more attention to the surgical report of the laparoscopic cholecystectomy, performed by an attending surgeon, stating that all spilled gallstones had been retrieved. We acknowledge that Gallstone retrieval cannot always be easily performed, due to varying surgical factors including the skills of the surgeon or his perseverance during the search. The overall incidence of spilled gallstones during laparoscopic cholecystectomy is 7.3% as stated in a retrospective literature review by Woodfield *et al*. It is estimated that 2.4% of these stones are not retrieved [3].

As only 0.08% to 0.3% of cholecystectomy-related complications are due to retained gallstones [4], the level of suspicion pertaining to spilled gallstones as a risk factor for postoperative complications is low. An iatrogenic gallstone spillage should be thoroughly documented; however, the intraoperative retrieval of spilled stones frequently does not follow a structured pattern and is mainly dependent on the surgeon. A previous cholecystectomy should always raise suspicion of intra-abdominal and extra-abdominal complications, especially on the right side of the trunk, independently of time since the procedure.

In case of gallstone spillage, we advise a systematic intraoperative exploration and adequate irrigation starting in the perihepatic region and Morrison’s Pouch following down the right paracolic gutter to the Pouch of Douglas. With respect to the positioning of the patient during the procedure, it also might be worthwhile to do a final overview in the Trendelenburg position to account for any backflow of sealed fluid or gallstone collections between intestinal or mesenteric folds.

## Conclusion:

We believe that a thorough, systematic intra-operative exploration and removal of spilled gallstones during cholecystectomy could reduce this particular long-term postoperative complication and suggest the exploration protocol described above should be mandatory in case of lost gallstones.
